# Microbial involvement in iodine cycle: mechanisms and potential applications

**DOI:** 10.3389/fbioe.2023.1279270

**Published:** 2023-10-30

**Authors:** Eva Duborská, Hana Vojtková, Michaela Matulová, Martin Šeda, Peter Matúš

**Affiliations:** ^1^ Faculty of Natural Sciences, Institute of Laboratory Research on Geomaterials, Comenius University in Bratislava, Bratislava, Slovakia; ^2^ Department of Environmental Engineering, Faculty of Mining and Geology, VŠB–Technical University of Ostrava, Ostrava, Czechia; ^3^ Radioactive Waste Repository Authority (SÚRAO), Praha, Czechia; ^4^ Department of Applied Chemistry, Faculty of Agriculture and Technology, University of South Bohemia, České Budějovice, Czechia

**Keywords:** iodine, bioreduction, biooxidation, biomethylation, bioremediation

## Abstract

Stable iodine isotopes are essential for humans as they are necessary for producing thyroid gland hormones. However, there are hazardous radioactive iodine isotopes that are emitted into the environment through radioactive waste generated by nuclear power plants, nuclear weapon tests, and medical practice. Due to the biophilic character of iodine radionuclides and their enormous biomagnification potential, their elimination from contaminated environments is essential to prevent the spread of radioactive pollution in ecosystems. Since microorganisms play a vital role in controlling iodine cycling and fate in the environment, they also can be efficiently utilized in solving the issue of contamination spread. Thus, this paper summarizes all known on microbial processes that are involved in iodine transformation to highlight their prospects in remediation of the sites contaminated with radioactive iodine isotopes.

## 1 Introduction

Iodine is a vital trace element essential for both humans and animals. Iodine deficiency disorders often occur in regions where the geochemical environment has low iodine levels. Iodine plays a crucial role in the synthesis of thyroid hormones, specifically T4 (tetraiodo-L-thyronine) and T3 (triiodo-L-thyronine) (Sorrenti et al., 2021). These hormones are responsible for regulating metabolism, growth, and development (Andersson and Braegger, 2021). They control basal metabolic rate, protein synthesis, long bone growth, and neuronal maturation (Mégier et al., 2023). Apart from its role in hormone synthesis, iodine deficiency is also a risk factor for thyroid cancer (Fan et al., 2021). Adequate iodine supply is crucial for preventing diseases of the mammary gland, as iodine lactones may protect against oxidative damage and inhibit cell division (Kupper and Gartner, 2007). Besides its biological importance iodine salts are widely used in pharmaceuticals (Nobukuni, 2009) and antiseptic agents (Grzybowski et al., 2018), radiocontrast agents (Pasternak and Williamson, 2012), light bulbs (Bregnhøj et al., 2019), solar cells (Sun et al., 2015), and LCD displays (Ma et al., 2011).

There are 36 known radioactive isotopes of iodine, with particular attention given to ^129^I and ^131^I (Petrov et al., 2022). Following the Fukushima power plant accident in 2011, the concentration of ^129^I in precipitation increased by four orders of magnitude (Xu et al., 2013), resulting in 100 times higher thyroid radioiodine concentrations of exposed cattle from the affected area in comparison to the control group (Horikami et al., 2022).

In offshore surface waters near Fukushima, ^129^I concentrations ranged from 0.002 to 0.133 pg. L^-1^ (Hou et al., 2013; Suzuki et al., 2013). Following the Fukushima nuclear power plant accident, ^129^I levels in the upper 100 m layer of seawater increased on average to 0.02 pg. L^-1^, with a maximum of 0.13–0.19 pg. L^-1^ (Suzuki et al., 2013; Xu et al., 2013). While the dominant iodine specie in seawater is iodate, Hou et al. (2013) observed that the primary form of ^129^I is iodide.

In the surface layers of Belarusian soil, ^129^I concentrations rose dramatically after the Chernobyl accident, increasing by three orders of magnitude, from 14.7 pg.kg^−1^ to 7.7 ng.kg^−1^ (Mironov et al., 2002).

The median value of yearly release of short-lived iodine isotopes from nuclear power plants in the United States is estimated to range from 10^–7^ to 10^–5^ GBq.GW^-1^. h^-1^, depending on the type of reactor (Ekidin et al., 2022).

Recently, the most promising materials for iodine remediation from nuclear waste include metal oxides (Muhire et al., 2022), bentonite (Yang et al., 2022) and zeolites (Zhang et al., 2022), bismuth-based mineral phases (Levitskaia et al., 2022), silica aerogel (Chang et al., 2022), organopolymeric structures (Kusumkar et al., 2021; Mohan et al., 2022) and various biomass-based sorbents, such as Bi-impregnated spent coffee ground biochar (Kwak et al., 2022). The application of microorganisms was also proposed (Shin et al., 2022).

**FIGURE 1:**
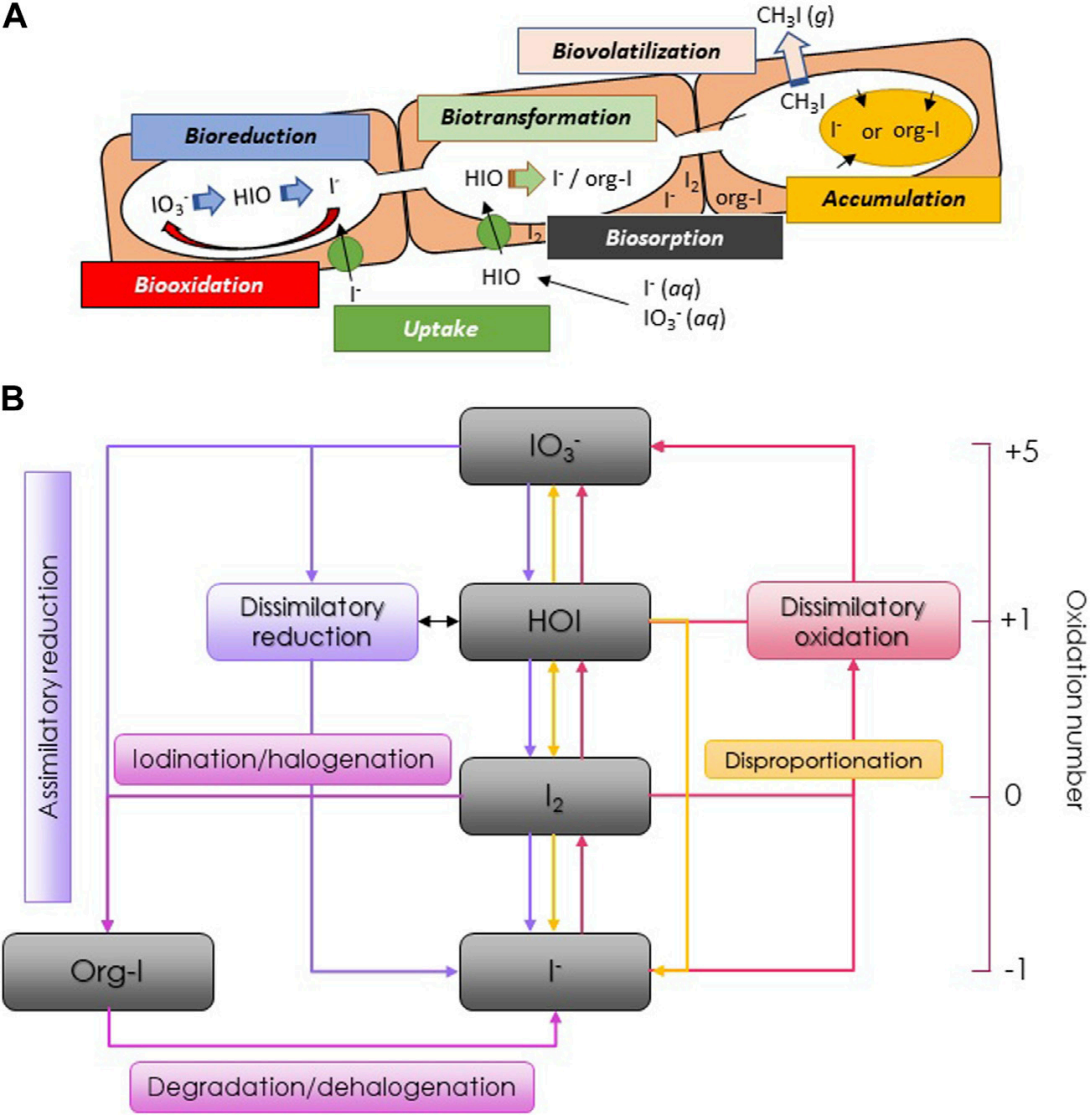
**(A)** Processes of microbially mediated iodine transformations; **(B)** Biochemical cycle of iodine.

Microorganisms play an important role in iodine cycling either by active production of its volatile species to the atmosphere or by changing environmental conditions in terrestrial system, thus affecting their retention. In aerobic environments, partially sorbed IO_3_^−^ species to sediments may be released back into the environment as a consequence of microbially generated reducing conditions (Guido-Garcia et al., 2015). On the other hand, extracellular oxidases of bacterial origin act as a catalyst for iodine retention in soil (Grandbois et al., 2023). Microbial influences on iodine speciation and its environmental fate are complex, governed by both extracellular and intracellular mechanisms, as depicted in Figure 1. Since this review focuses on the biochemical transformations of iodine and their potential in remediation strategies, for comprehensive information on the global iodine environmental cycle refer to our previous work (Duborská et al., 2021a; Duborská et al., 2021b).

## 2 Microbial reduction of iodate

Determining iodine species in surface waters poses a significant challenge; iodate (IO_3_^−^) is noted as the prevailing species, which can undergo microbially mediated reduction (Zaruba et al., 2017; Wadley et al., 2020). The exact mechanism of iodate reduction is still unclear; however, available data suggest that it is mediated by microorganisms via enzymatic transformations and interactions with extracellular metabolites. It was widely assumed that iodate can be reduced aerobically in surface waters to iodide by microorganisms having a nitrate-reducing activity, such as *Escherichia coli* (Tsunogai and Sase, 1969). It was hypothesized that iodate electron transport pathways are terminated with the reduction of iodate by nitrate reductase, which serves as an alternative electron acceptor (Toporek et al., 2019). Later, however, Mok et al. (2018) demonstrated that reduction of IO_3_^−^ by *Shewanella oneidensis* does not involve nitrate reductase and it is independent of nitrate uptake. On the other hand, Kengen et al. (1999) isolated a chlorate reductase resembling nitrate reductase, which possessed nitrate, iodate and bromate reducing activity. Lee et al. (2018) isolated a bacterium from the groundwaters of radioiodine contaminated Hanford site, which is closely related to *Agrobacterium* sp. and was capable of the simultaneous reduction of nitrate and iodate. Furthermore, iodate reduction was not observed when nitrate was absent from the growth medium.

Besides bacteria, several species of phytoplankton are also producing iodide, as reported by Bluhm et al. (2010), who assumed that due to increased cell permeability during the senescent phase of the algae, the exuded sulfidic species reduced the iodate. The reduced sulfidic species or glutathione were also found to promote iodate reduction during cell apoptosis (Taurog et al., 1966). However, later, it was suggested that the presence of iodide may be the result of the release of I^−^ from C-I bonds during the decomposition of organic matter instead of IO_3_^−^ reduction by sulfide (Farrenkopf et al., 1997).

The idea of the existence of an independent iodate reductase was already proposed by early studies, however, such enzyme has not been yet completely characterized. Iodate-reducing bacterium *Pseudomonas* sp., isolated from marine sediments by Amachi et al. (2007), reduces iodate anaerobically via a specific iodate reductase. Recent studies also indicate that iodate reduction by *S*. *oneidensis* involves a yet unidentified iodate reductase associated with the outer membrane MtrAB module, which enables the extracellular reduction of iodate (Toporek et al., 2019). The same bacterial specie was also studied by Shin et al. (2022), who suggested that an extracellular EEC-associated dimethyl sulfoxide (DMSO) reductase with a molybdenum enzyme center is responsible for formate-dependent iodate reduction. A recent study by Reyes-Umana et al. (2022) showed that the dissimilatory iodate reduction by estuarine bacterium *Denitromonas* sp. is mediated by a molybdenum-dependent iodate reductase. Such molybdoenzyme, a dimethylsulfoxide reductase, was also isolated from *Pseudomonas* sp. (Yamazaki et al., 2020). This specie uses iodate as a terminal electron acceptor for anaerobic respiration. Based on their results with hypobromous acid, Müller et al. (2021) suggested that the reaction of dimethyl sulfide (DMS) with hypoiodous acid might also be a possible mechanism of assimilatory iodate reduction.

## 3 Microbial oxidation

Shimada et al. (2022) presented, that 90% of ^129^I in drain water of the Fukushima nuclear power plant was in the form of iodide. Still, it can be oxidized by microorganisms. An iodine-oxidizing bacterium, *Pseudomonas iodooxidans*, was first identified by Gozlan and Margalith (1974). This specie possesses iodide oxidation abilities due to the production of a specific heme-peroxidase. Such enzymatic activity requires oxygen, but not hydrogen peroxide, as an electron acceptor. This was proposed by Amachi et al. (2005b) after isolating iodide oxidizing bacteria from natural gas brine waters, which were phylogenetically most closely related to *Roseovarius tolerans* and *Rhodothalassium salexigens*. This enzyme was later identified as a multicopper oxidase by Suzuki et al. (2012), who isolated it from an *Alphaproteobacteria* strain.

The molecular iodine produced by catalytic oxidation of iodide by *Roseovarius* sp. inhibited the growth of other sensitive bacteria in the environment, such as *E*. *coli*, as demonstrated by Zhao et al. (2013). Later, Yuliana et al. (2015) suggested that this iodide oxidizing enzyme could be utilized as a novel antimicrobial enzymatic system.

A newly discovered aerobic iodide oxidizing bacteria *Iodidimonas gelatinilytica* isolated by Iino et al. (2021) from brine and surface seawater was capable of oxidizing iodide by a putative multicopper oxidase, which is phylogenetically distinct from other bacterial multicopper oxidases (Amachi and Iino, 2022).

In terrestrial systems, extracellular iodide oxidases of bacterial and fungal origin have an important role in iodine retention potential of surface soils (Seki et al., 2013). In early studies, it was suggested by Raciborski (1905) that *Aspergillus niger* produces an extracellular iodide-oxidase, which is responsible for the oxidation of iodide to free iodine. Later, Pearce (1940) discovered that this oxidation is catalyzed by hydrogen peroxidase, therefore, the oxidation of iodide is a secondary oxidation reaction by hydrogen peroxide formed as a result of oxidation of glucose catalyzed by glucose oxidase. Similar multicopper oxidase was found to play role in terrestrial bacterium *Rhodanobacter denitrificans* (Shiroyama et al., 2015).

In soils, at least part of iodide is oxidized by fungal laccase in the presence of redox mediators (Nihei et al., 2018; Lee et al., 2020). Nihei et al. (2018) isolated iodide-oxidizing fungi from soil, including the strains of *Trichoderma hamatum* and *Scytalidium album*.

## 4 Iodine biomethylation

Methylated iodine production is a growth rate-dependent process in most marine heterotrophic bacterial groups (Gómez-Consarnau et al., 2021). Iodide-oxidizing bacteria also possess volatile organic iodine production abilities. Fuse et al. (2003) identified iodine-methylating marine bacterium (phylogenetically close to *R*. *tolerans*) that produces CH_2_I_2_, CH_3_I and CH_2_ClI. The sole production of CH_3_I by this specie was reported by Amachi et al. (2005b). Methyl iodide production was observed in members of *Proteobacteria*, *Cytophaga-Flexibacter-Bacteroides* groups (Amachi et al., 2004), *Alteromonas* sp., *Vibrio* sp. (Amachi et al., 2001), and marine cyanobacterium *Calothrix parasitica* (Okuda et al., 2023). Even brown algae *Ectocarpus* sp. (Küpper et al., 2018) and *Laminaria* sp. (Nightingale et al., 1995) produce methylated iodine compounds.

Terrestrial bacteria, e.g., *Varivorax* sp. (Amachi et al., 2001), and microscopic filamentous fungi, such as *Alternaria alternata* and *Fusarium oxysporum* (Ban-nai et al., 2006; Duborská et al., 2017), are also capable of iodine methylation.

Although, the exact mechanism is not yet completely explained, iodine methylation by microorganisms is probably following the pathway of the Challenger mechanism that is typical for As methylation (Challenger, 1951). Alternatively, a vanadium-dependent haloperoxidase enzyme is involved in this unique process (Punitha et al., 2018).

## 5 Bioaccumulation

Various elements, including radionuclides, can be actively taken up by microorganisms (Urik et al., 2010; Urik et al., 2011) or be fixed to microbial cell walls or extracellular polysaccharides of biofilms (Čerňanský et al., 2007; Littera et al., 2011). Iodide accumulation in bacteria presumably takes place via nucleophilic substitution of functional groups in cellular organic molecules (Li et al., 2011). The iodine uptake ability is relatively high in *Cyanobacteria*, green algae and *Ochrophytes* (Fukuda et al., 2014). High accumulation ability was found in bacterial strains of *Streptomyces/Kitasatospora* spp., *Ralstonia/Cupriavidus* spp., and *Bacillus mycoides* that were isolated from aquifers at ^129^I-contaminated Savannah River site. However, these aerobic bacteria did not accumulate significant amounts of iodine (Li et al., 2011). Fukuda et al. (2014) identified three cyanobacteria (*Nostoc commune, Scytonema javanicum, Stigonema ocellatum*) and xantophycean algae *Ophiocytum* sp. with high ability to accumulate radioactive ^125^I from water. Amachi et al. (2005a) isolated two iodide-accumulating bacteria from marine sediments belonging to the *Flavobacteriaceae* family and were closely related to *Flexibacter aggregans* and *Arenibacter troitsensis*. Iwamoto and Shiraiwa (2012) suggested that microalgae *Emiliana huxleyi* could be used in industry for extracting iodine from iodine-contaminated waters, since it is able to accumulate ten times more iodine than normally found in seawaters.

According to our best knowledge, only two studies available present iodine accumulation rates of microscopic filamentous fungi. Therefore more attention should be paid to these species since they cover up to 30% of soil microbial biomass (Gschwend et al., 2022) and participate in a whole range of processes (Costa et al., 2018).

Korobova (2010) reported 1.8–147 mg.kg^-1^ iodine levels in further not specified soil microorganisms (dry weight) in the central Russian plain but suggested that fungi might have been a great contributors to this number. The highest iodine accumulation rates under laboratory conditions were reported for *Alternaria alternata*, a soil-borne pathogen, up to 63 μg.g^-1^ with an equally high capacity for both iodide and iodate species from culture media (Ban-nai et al., 2006; Duborská et al., 2017). *Penicillium chrysogenum* is able to accumulate up to 10.2% of iodine depending on soil iodine content and the degree of the organisms' tolerance to its high concentrations (Letunova et al., 1986).

## 6 Potential use of microorganism in bioremediation

Iodine remediation techniques involving microorganisms in contaminated sites have been studied in limited case studies, however, they offer great potential for addressing iodine contamination through various interaction mechanisms. These mechanisms include microbial methylation of iodide to volatile gases and microbial reduction of iodate to iodide (Shin et al., 2022). Understanding and harnessing these microbial processes can significantly contribute to effective iodine remediation strategies. Species which could be potentially useful for iodine remediation are presented in Table 1.

**TABLE 1:** Species potentially capable of iodine remediation.

Species	Mechanism	Concentration		Substrate I concentration	Reference
Microalgae					
*Chlorella sorokiniana*	Accumulation	1,200 μg.g^-1^		Not provided	Gómez-Jacinto et al. (2012)
*Mediopyxis helysia*	Volatilization	Emission rates [pmol.min^-1^m^-2^]		Not provided	Thorenz et al. (2014)
		CH_3_I	0.32–0.8		
		CH_2_ICl	0.04–0.22		
		CH_2_I_2_	0.21–0.50		
*Porosira glacialis*		CH_3_I	0.21–0.69		
		CH_2_ICl	0.02–0.22		
		CH_2_I_2_	0.27–0.44		
*Tisochrysis lutea*	Accumulation, bioreduction	1,477 ± 87.37 µg^a^		5,000 µM I^−^	van Bergeijk et al. (2016)
		1,613 ± 337.1 µg^a^		2,500 µM IO_3_^−^	
*Phaeodactylum tricornutum*		1,077 ± 75.06 µg^a^		5,000 µM I^−^	
		495 ± 19.9 µg^a^		2,500 µM IO_3_^−^	
*Dunaliella salina*		1713 ± 998.1 µg^a^		5,000 µM I^−^	
		900 ± 180 µg^a^		2,500 µM IO_3_^−^	
*Emiliania huxleyi*		Up to 4.4 µM		1 M IO_3_^−^	Iwamoto and Shiraiwa (2012)
Further unidentified freshwater microalgal biofilm		350 ± 29 mg.kg^-1^		Not provided	Han et al. (2016)
Aerobic bacteria					
*Frexibacter aggregans*	Accumulation	220 pM^a^		Not provided	Amachi et al. (2005a)
*Arenibacter troitsensi*		0.1 µM^a^			
Deuteromycetes					
*Alternaria alternata*	Volatilization	0.07%		0.18–0.22 µg.L^-1^	Ban-nai et al. (2006)
		11.2%–18.7 %			
		11.2%–18.7 %		1 mgL^-1^	Duborská et al. (2017)
	Accumulation	0.12 %		0.18–0.22 µg.L^-1^	Ban-nai et al. (2006)
		32.7%–37 %		1 mgL^-1^	Duborská et al. (2017)
*Cladosporium Cladosporioides*	Volatilization	0.12 %		0.18–0.22 µg.L^-1^	Ban-nai et al. (2006)
		7.1%–15.5 %		1 mgL^-1^	Duborská et al. (2017)
	Accumulation	37 %		0.18–0.22 µg.L^-1^	Ban-nai et al. (2006)
		2.9%–3.9 %		1 mgL^-1^	Duborská et al. (2017)
Ectomycorrhyzal fungi					
*Cemococcum geophilum*	Volatilization	18 ± µg.g^-1^		20 mM	Redeker et al. (2004)

^a^ dry weight.

*Deinococcus radiodurans*, a radiation-resistant bacterium known for its remarkable resilience, has shown promising results as an excellent candidate for on-site bioremediation. Research conducted by Choi et al. (Choi et al., 2016; Choi et al., 2017; Shim et al., 2018) demonstrated that the biomass of *D. radiodurans*, containing biogenic gold and silver nanoparticles, prepared by bioprecipitation, exhibits more than 99% removal efficiency. This suggests that these complexes hold significant potential for addressing iodine contamination.

In a recent study conducted by Tang et al. (2022), a novel approach for capturing radioactive iodine from polluted atmospheres was presented. The researchers developed a porous sponge-like complex absorbent composed of alginate and fungal mycelium of *Actinomucor elegans*. This absorbent material offers a high surface area and efficient capture of radioactive iodine, providing a potential solution for addressing airborne contamination.

Nanocomposites made of bacterial cellulose pellicles present another viable approach for iodine capture. These nanocomposites, which exhibit irregularly webbed and highly hydrated structures, offer superior chemical and structural stability. Cellulose-producing bacteria, such as *Acetobacter xylinus*, are responsible for producing these nanocomposites. Zia et al. (Zia et al., 2022) explored the potential of bacterial cellulose pellicle nanocomposites as effective iodine capture agents from both vapor and aqueous solutions. These nanocomposites’ have high surface area and their stability make them suitable for capturing and removing iodine contaminants.

Au-Duong and Lee (2018) developed a flexible metal-organic framework-bacterial cellulose nanocomposite by immersing bacterial cellulose pellicles in zinc ions and 2-methylimidazole. After photothermal regeneration, this nanocomposite exhibited remarkable performance, maintaining 99% and 87% of its initial iodine uptake capacity during the second and sixth uses, respectively. This finding highlights the potential of metal-organic framework-bacterial cellulose nanocomposites for long-term and sustainable iodine remediation efforts.

Sasamura et al. (2023) proposed *Azocarus* sp. as a potential bioaugmentation agent for ^129^I-contaminated waters. This strain demonstrates an effective iodate respiration mechanism, making it a promising candidate for bioremediation in water environments.

In addition to these innovative approaches, iodine biomethylation holds promise as a means for capturing and recovering iodine volatilized by microorganisms. Technologies such as soil vapor extraction can be employed to capture iodine vapors, while activated carbon adsorption provides an effective method for capturing and concentrating iodine from both air and water sources. Strickland et al. (2017) explored the use of activated carbon as a reliable medium for iodine capture, which can subsequently be regenerated or safely disposed of, ensuring the sustainability of the remediation process.

## 7 Conclusion

The anthropogenic input of radioactive iodine into the atmosphere is a matter of great significance. Currently, there is considerable focus on the development of environmentally friendly adsorbents. Biomass-based sorbents show promise as a cost-effective solution for remediating iodine from water contaminated with atmospheric deposits of radioactive iodine, thereby preventing its incorporation and biomagnification in the food chain. The contamination of seawater and kelp farms with radioiodine poses a particular threat, as these species have been found to accumulate high levels of radioactive ^129^I. While the study of native soil microbiota should be taken into consideration, the precise role and mechanisms by which they retain and release iodine from soils remain unclear. Nevertheless, soil serves as a significant sink for anthropogenic iodine from the atmosphere, which can subsequently accumulate in plants and forage.

The studies discussed in this paper provide valuable insights into microbially-involved iodine remediation techniques, covering microbial interactions, bioaugmentation, nanocomposites, and biomethylation. By understanding and utilizing these diverse strategies, researchers can contribute to the development of effective and sustainable iodine remediation approaches. Microorganisms play diverse roles in the cycling of iodine and offer promising potential for bioremediation strategies. Further research is necessary to fully comprehend the underlying mechanisms and explore practical applications of microbial processes in addressing iodine contamination. These findings open up new possibilities for the development of effective and sustainable approaches to mitigate iodine pollution in various environments.
